# The Application of Electro- and Magneto-Encephalography in Tinnitus Research – Methods and Interpretations

**DOI:** 10.3389/fneur.2014.00228

**Published:** 2014-11-13

**Authors:** Peyman Adjamian

**Affiliations:** ^1^MRC Institute of Hearing Research, Nottingham, UK

**Keywords:** tinnitus, magnetoencephalography, electroencephalography, induced and evoked responses, source localization, top-down processing, functional and effective connectivity

## Abstract

In recent years, there has been a significant increase in the use of electroencephalography (EEG) and magnetoencephalography (MEG) to investigate changes in oscillatory brain activity associated with tinnitus with many conflicting results. Current view of the underlying mechanism of tinnitus is that it results from changes in brain activity in various structures of the brain as a consequence of sensory deprivation. This in turn gives rise to increased spontaneous activity and/or synchrony in the auditory centers but also involves modulation from non-auditory processes from structures of the limbic and paralimbic system. Some of the neural changes associated with tinnitus may be assessed non-invasively in human beings with MEG and EEG (M/EEG) in ways, which are superior to animal studies and other non-invasive imaging techniques. However, both MEG and EEG have their limitations and research results can be misinterpreted without appropriate consideration of these limitations. In this article, I intend to provide a brief review of these techniques, describe what the recorded signals reflect in terms of the underlying neural activity, and their strengths and limitations. I also discuss some pertinent methodological issues involved in tinnitus-related studies and conclude with suggestions to minimize possible discrepancies between results. The overall message is that while MEG and EEG are extremely useful techniques, the interpretation of results from tinnitus studies requires much caution given the individual variability in oscillatory activity and the limits of these techniques.

## Introduction

The precise neural mechanism of subjective tinnitus generation is unknown but it is a symptom of many pathologies. A general conception is that damage to the auditory periphery (mainly the cochlea) results in functional modifications of the central auditory system. According to the definition by Jastreboff (1): “Tinnitus is the perception of a sound that results exclusively from activity within the nervous system without any corresponding mechanical, vibratory, activity within the cochlea, and unrelated to external stimulation.” According to this, Jastreboff argued that tinnitus involves not merely abnormalities of cochlear function but abnormal tinnitus-related signals within the nervous system. Subsequently, numerous animal studies have provided support for this hypothesis, reporting changes in neural activity within structures of the central auditory system (2–7). Therefore, fundamental to the concept of subjective tinnitus is the idea that the central nervous system is involved in the perception of the sound and the associated emotional and psychological symptoms.

In human beings, several clinical and psychoacoustic observations suggest that while damage to the auditory periphery can trigger tinnitus, it is not by itself sufficient to sustain it, and that tinnitus has neural correlates in the brain (8). Furthermore, neuroimaging studies such as functional magnetic resonance imaging (fMRI) have revealed abnormalities at various stages of the classical auditory pathway, including the inferior colliculus (9) and the auditory cortex (10, 11). Hoke et al. (12) were the first to employ magnetoencephalography (MEG) and measure the evoked response to a 1-kHz tone in participants with tinnitus. Since then there has been a range of studies using both MEG and electroencephalography (EEG) to investigate oscillatory changes in tinnitus compared to non-tinnitus controls to gain a better understanding of the central mechanism of this disorder [see Adjamian et al. (13) for a review]. However, the results have been largely contradictory. There are potentially many reasons for the discrepancies, which may arise from variability between participants’ clinical characteristics, the inappropriate samples, and comparison groups used, but also the application and limitation of M/EEG techniques as well as the misinterpretation of findings. In the light of recent renewed interest in application of these techniques in tinnitus research, it is important to be aware of how these techniques reflect brain function and malfunction.

But how can these techniques be best applied to tinnitus research, what are their limitations, and how easy is it to misinterpret the results? A rudimentary description of M/EEG techniques for the tinnitus audience is helpful to contextualize the findings from studies that employ these techniques. In this article, I intend to highlight the strengths and limitations of M/EEG for tinnitus research that have potential implications for interpreting results as well as some misconceptions with the aim of generating discussion. I will start with a brief description of the sources of the M/EEG signals and what these represent. I will also discuss some pertinent issues regarding the various types of M/EEG data and a general introduction to relevant analysis techniques that have been applied in tinnitus research. This article is not intended to provide a detailed account of the literature in any of the areas discussed, nor is it meant to be a comprehensive review of recent M/EEG studies in tinnitus.

### Why M/EEG?

So why should we use M/EEG to study tinnitus? What is the advantage of these techniques for tinnitus research compared to animal studies or other neuroimaging techniques such as fMRI? The main strength of M/EEG over other imaging techniques such as fMRI is that the signals are direct measurements of neural activity as they reflect real-time information transfer between neurons. This dynamic activity is recorded with millisecond temporal resolution so that precise information about the timing of neural events can be obtained in human beings. Furthermore, temporal relations between distributed neural ensembles can be assessed only with M/EEG as they provide wide spatial sampling, covering the entire brain. The recorded oscillatory activity conveys neuronal processing that can be used to measure effective and functional connectivity between disparate brain regions to examine network models of tinnitus. Finally, M/EEG data can be recorded in total silence. In general, auditory research with fMRI presents significant challenges as the MR scanner generates an acoustic sound exceeding 100 dBA. The silent environment of M/EEG is important for tinnitus research since it is not known how the MRI scanner noise can interfere with tinnitus-related brain activity.

With regard to the animal models of tinnitus, the applicability of results from animal studies to human beings is limited, partly because of the restricted spatial sampling and partly because of the difficulties in assessing the presence of tinnitus and associated distress in laboratory animals. Given these challenges, M/EEG allows us to undertake a proportion of investigations non-invasively in human participants and observe tinnitus-related neural changes directly in tinnitus patients, coupled to more reliable assessment of tinnitus characteristics and comorbidities. More importantly, as tinnitus is likely to emerge from abnormal activity of distributed brain networks, M/EEG allow us to examine direct neural activity, simultaneously from numerous brain regions, as well as their large-scale interaction. Thus, given their excellent temporal resolution, wide spatial sampling, and ever improving spatial resolution, M/EEG present an excellent opportunity to investigate the central mechanism of tinnitus in human beings that is unrivaled by other neuroimaging techniques.

### The proposed central mechanisms of tinnitus

It is not within the remit of the current discussion to review the various putative mechanisms of central tinnitus generation as these have been discussed at length elsewhere (13–15). Here, I briefly mention those which can be examined non-invasively with M/EEG and which become relevant to the discussion later in this article.

Within the structures of central auditory system, different types of abnormal activity have been identified, mainly based on animal studies. It is generally believed that deafferentation due to hearing-loss triggers a chain of neural events that lead to hyperactivity and the tinnitus sensation (14). These neural events include: increase spontaneous firing of neurons’ spontaneous activity, changes in temporal firing pattern, increased synchrony between neurons, and reorganization of the tonotopic map due to deafferentation following hearing loss (16). Human brain imaging can reflect these neural events at the macroscopic level, and therefore the findings in neuroimaging studies of tinnitus in human beings is commonly interpreted as resulting from hyperactivity, or increased neural synchrony (17). Changes in the amplitude of M/EEG oscillations can be interpreted as either changes in cortical areas responding to a specific stimulus or of the amount of neuronal synchrony within or between neural ensembles.

Llinás et al. (18) have proposed a model of altered thalamo-cortical rhythms to explain the neural mechanism of various abnormalities associated with a range of neurological conditions including Parkinson’s disease, neurogenic pain, depression, and tinnitus. According to this thalamo-cortical dysrhythmia (TCD) model, tinnitus is due to a disruption of activity between thalamus and cortex initiated by neural deafferentation, due to hearing loss, which causes inhibition of thalamic neurons. This in turn leads to changes in oscillatory activity at the cortical level and large-scale slow-wave and gamma activity in the neighboring cortical regions. This model proposes a number of specific tinnitus-related changes in oscillatory activity, namely increase delta/theta activity in a localized region surrounded by gamma activity. These predictions can be assessed with M/EEG but as will be described later, results are not entirely supportive of the model.

Rauschecker et al. (19) have proposed the gating mechanism of tinnitus according to which the appearance of tinnitus depends on individual differences in the effectiveness of the noise-cancelation system mediated by structures within non auditory regions. The model hypothesizes that under normal circumstances hyperactivity in auditory pathways is canceled out at the level of the thalamus by an inhibitory feedback loop (noise-cancelation system) originating in the ventromedial pre-frontal cortex and the nucleus accumbens. These structures are part of a circuit in the subcallosal ventral striatum, which identify the presence of unwanted neural activity (in this case tinnitus). The unwanted neural activity is fed back to the reticular nucleus of the thalamus, which removes the signal from input to the auditory cortex. Support from this model has come from analysis of structural images of the brain (20–22) but overall the anatomical studies provide scant support for the gating mechanism (23) and functional imaging studies are even less convincing. The proposed anatomical structures involved in this model reside deep in the brain and thus whether M/EEG can be reliably used to assess their activity is debatable for reasons that will be discussed in the next section.

The basic theoretical assertion of the various network models is that altered activity in the central auditory pathways is insufficient to give rise to phantom percepts such as pain and tinnitus. For tinnitus to be consciously perceived, auditory activity requires integration with activity from the global awareness or attentional network (24) involving frontal and parietal areas. Moreover, the neural mechanisms associated with the tinnitus percept are distinct from those responsible for tinnitus-related distress, which requires the activation of the distress network (24). Bothersome tinnitus is thus continuously associated with aversive emotional states, involving abnormal input from structures of the limbic system, including the amygdala and the insula. Accordingly, this may partly explain why many people with hearing loss and concomitant increase in activity in auditory pathways do not develop tinnitus. Numerous M/EEG studies have attempted to examine the various assumptions of such models with varying degrees of success, which will be discussed in Section “The Role of Non-Auditory Brain Regions in Tinnitus.” The network models of tinnitus can also be examined by means of frequency coupling to assess inter-regional connectivity, which is discussed in Section “Measures of Brain Connectivity.”

## The Physiological Basis of M/EEG Signals

MEG and EEG are extremely sensitive to miniscule changes in the magnetic fields and electrical potentials, respectively, which are produced by changes in the electrical activity within the brain. Neural activation generates an electric current, which corresponds to the electric potentials in case of EEG, and magnetic fields in case of MEG, measured outside the head with an array of electrodes.

There are two main types of neuroelectric events associated with the activity of neurons in the brain: the action potential and the postsynaptic potential. While action potentials can be large in amplitude (70–110 mV), they do not fire with sufficient synchrony, and their time course is relatively short (~1 ms) and hence signal summation is unlikely. On the other hand, the postsynaptic potentials (PSPs) have longer time courses lasting for several tens, or even hundreds, of milliseconds with a peak value of ~10 mV (25), which facilitates summation of synchronized activity from adjacent neurons. M/EEG signals are thus thought to arise from changes that occur in the resting membrane potential of the dendritic trees of the cortical pyramidal neurons (26) caused by synaptic input, the excitatory postsynaptic potential (EPSP), and the inhibitory postsynaptic potential (IPSP). The changes in the resting potential are due to the exchange of ions between the inside and outside of the neuron, which causes current flows both in the extracellular and the intracellular space, through the volume conductor (27). The extracellular currents (also known as secondary or volume currents) spread through the brain, pass through the tissues, and the skull, producing voltage potentials that vary in amplitude over the scalp and are measurable with EEG electrodes. The magnetic field is generated by both the extracellular volume currents and intracellular currents (also known as primary currents) (28, 29). M/EEG oscillations thus reflect the oscillatory macroscopic local field potentials (LFPs) from intracranial recordings, retaining the same millisecond temporal resolution as LFPs, with the difference being that the latter are observed at higher spatial resolution.

M/EEG are primarily sensitive to superficial sources in the neocortex, which consist of two major type of neurons: stellate cells and pyramidal cells (30). Pyramidal cells are elongated structures possessing an apical dendrite while stellate cells are more rounded with dendrites that are equally spread around the cell body. Stellate cells are a type of “interneurons,” which mainly exist to make short connections between neurons. Lorente de No [1947, cited in (26)] differentiated between “open field” and “closed field” neurons. Stellate cells are examples of “closed field” neurons as they have relatively symmetric dendrites and thus their fields cancel out each other. On the other hand, pyramidal cells have asymmetrical dendrites in parallel to each other and therefore generate dipole-like potentials, which could be recorded at a distance. Signal summation requires that neurons are regularly arranged and have a measurable M/EEG signal. Pyramidal cells vary in height from about 10 μm up to 100 μm and are spatially organized in columns so that the axes of their dendritic trees are parallel with each other and oriented toward the cortical surface (31). Therefore, both spatial and temporal summation of their activity is possible when sufficient synapses are active in relative synchrony (32).

The detection of a large enough amplitude M/EEG signal requires spatial and temporal synchrony of a large mass of cortical neurons. Synchronous sources produce much larger signals than asynchronous sources and optimal signal summation occurs for PSPs with zero time lag (33). M/EEG detect summed activities of 10,000 to 50,000 synchronously active neurons (26). Nunez (34) has estimated that in order to obtain amplitude of a scalp EEG signal, the synchronous activity of neurons within a 1 cm^2^ cortical surface is required.

**Figure 1:**
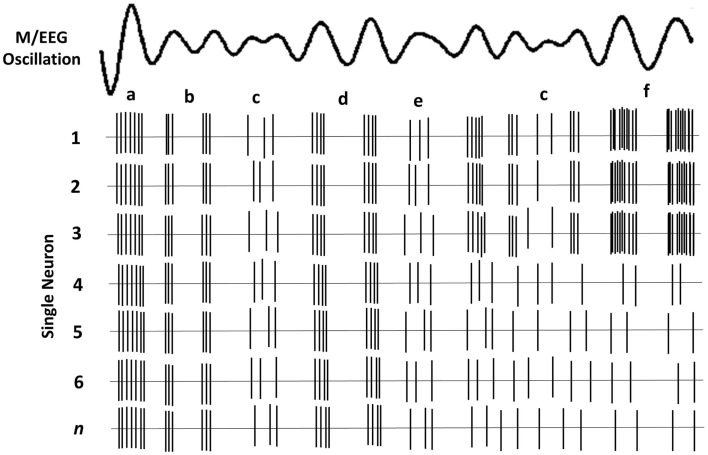
**Schematic of oscillatory activity due to firing of a hypothetical neuronal ensemble**. Each row represents the activity of a different neuron. (a) All neurons fire in synchrony at a relatively high rate. This coherent firing rate gives rise to a large amplitude LFP signal; (b) lower rate coherent firing gives rise to a lower amplitude component of the oscillatory LFP; (c) stochastic neural firing, where only some neurons fire coherently, results in much lower amplitude in the oscillatory signal; (d) lower firing rate of all neurons gives rise to lower amplitude signal; (e) lower amplitude oscillation when only some neurons have coherent firing; (f) fewer neurons fire but their high rate of coherent firing gives rise to a similar amplitude as *a*.

The brain’s rhythmic oscillatory activity is entrained by factors related to neuronal assemblies and include the following (35): (i) the intrinsic properties of the neuronal membrane, (ii) the structure of the interconnectivity between network elements and synaptic processes related to the function of feedback and feed-forward (e.g., thalamo-cortical and cortico-cortical) loops, and (iii) the modulating effects of neurotransmitters. The signal measured on the scalp is the spatial average of potentials produced by the underlying neuronal concentration. Therefore, M/EEG amplitude in each frequency band can be related to either the synchrony of the underlying current sources, and/or the extent of the area (total number of neurons) activated (33). It thus follows that a reduction in amplitude is a desynchronization of current sources (36), which in theory, occur as a result of either reduction in source magnitude or reduction in the activated surface area (33). M/EEG do not have the required resolution to determine whether a particular amplitude is due to increased synchrony within a neural population or to an increase in total number of activated neurons. Figure 1 depicts a simplified schematic of the effect of various types of synchrony in neural firing and the resulting M/EEG oscillatory activity.

### Differences between MEG and EEG

Despite their many similarities, EEG and MEG have certain important differences, which have been discussed extensively elsewhere (37–39). The possibility that contradictory results from M/EEG studies in tinnitus are at least partly due to differences between MEG and EEG cannot be discounted. Here, I will briefly describe those differences that may become important when interpreting and comparing the results between studies that employ MEG and EEG.

Each pyramidal neuronal column behaves as an electrical dipole; however, not all active brain regions produce detectable scalp fields and potentials. EEG is sensitive to dipoles oriented in any direction but is more sensitive to the radially oriented dipole when both radial and tangential dipoles are present (33). However, because the EEG signal is highly dependent on volume conduction, the signals are smeared as they pass through the surrounding tissue and the poorly conductive skull. The EEG measures the difference in voltage, or potential, between two electrode sites across the scalp one of which is placed at an electrically silent location to act as a reference point. The problem with reference points is that no electrode site is completely electrically silent (37). Therefore, absolute power and phase of scalp EEG signal in a given frequency band is ambiguous because the results are strongly influenced by the activity at the reference electrode (40). The surface distribution of currents is also somewhat distorted as the conductivity and the thickness of the skull is non-uniform. Thus estimating the strength and distribution of the signal at the EEG sensors on the scalp (forward solution) is more complex (41) and the estimation of signal sources (inverse solution) is less precise (42). EEG potentials cannot be considered a true reflection of the brain’s electrical events, contributing an estimated 5% to the extracranial measured magnetic field only (43). The magnetic fields recorded by MEG, however, are significantly less distorted by the surrounding tissue, particularly within a spherically homogenous head model whereby the secondary volume currents diminish rapidly. Thus the magnetic field recorded outside the head reflects the intracellular primary currents and are essentially the same as what would be recorded if the brain surface were exposed (29). A simple magnetometer measures the absolute magnitude of the magnetic field without the need for a reference point. Because of this, the forward solution is far easier to obtain with MEG than with EEG, and hence signal source reconstruction (inverse solution) tends to be more accurate with MEG (39, 44).

Electroencephalography is more sensitive to signals arising from sub-cortical structures while MEG is most sensitive to the superficial cortical sources (37, 45). Goldenholz et al. (45) compared signal-to-noise ratio (SNR) of cortical sources between MEG and EEG. The SNR of deep sources for EEG was larger compared to MEG, whereas for superficial sources, SNR was higher for MEG. Therefore, EEG is more likely to measure activity of sub-cortical structures, however, it should be noted that detecting a signal is not the same as its accurate localization. Localization of deep sources with EEG is complicated due to the afore-mentioned uncertainties in the forward solution.

Another difference is that MEG is sensitive to mainly tangential sources while EEG can detect both tangential and radial sources. When using a spherically symmetric volume conductor to model the intracranial source of a magnetic field, only the tangential sources are measurable because theoretically, in a perfectly symmetrical volume conductor radially oriented sources do not produce externally measureable magnetic fields (37, 46).

**Figure 2:**
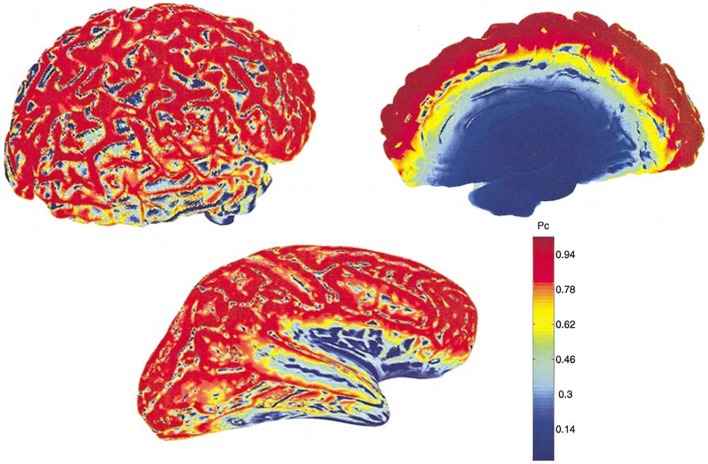
**MEG detection probability maps of entire brain for one person by Hillebrand and Barnes (**47). Data were recorded with a 151 MEG scanner with third order gradiometer configuration. Radial and tangential sources are on the cortical gyri and sulci, respectively. Much of the sensory cortex is on the sulci and can be detected by MEG. Note that the probability of detecting signal sources decreases with depth [adapted from Hillebrand and Barnes (47)].

This theoretical insensitivity of MEG to radial sources requires further clarification because this is not strictly true in practice. MEG measures magnetic fields that arise from the intracellular or extracellular currents depending on the orientation of the measurement coils relative to the head. If the axis of the coil is perpendicular to the head such that it measures only the radial component of the extracranial magnetic field, the measured magnetic field will be just the intracellular currents. But if the axis of the measurement coil is tilted relative to the head, it measures the tangential component of the magnetic field, which will reflect both the intracellular and the extracellular current flows. In practice, the coils are perpendicular to the head and thus measure the intracellular currents, but with the multi-channel systems, many of the channels are tilted relative to the head. Hillebrand and Barnes (47) estimated the detection probability of MEG across the entire brain that is visible to MEG sensors and showed that MEG is not completely insensitive to radially oriented sources. They found that only 5% of the whole cortical surface is within 15° of radial sources and that it is source depth, rather than orientation, that compromises the sensitivity of MEG to activity in the brain. Thin strips of only ~2 mm wide at the crest of gyri are poorly resolvable by MEG but as these strips are adjacent to tangential sources, they become detectable by the MEG sensors [(47); see Figure 2]. More recently, Ahlfors et al. (48) have quantified the dependency of both MEG and EEG on source orientation and found that, in general, MEG is insensitive to radial sources while EEG is sensitive to all components; however, as with Hillebrand and Barnes (47), they found that only few cortical sources have the precise orientation that renders them silent to MEG.

The differential sensitivity of MEG and EEG to source orientation has important practical implications that could underlie some of the observed discrepancies between studies, not only in tinnitus but in other areas of brain research. Potentially, this problem is accentuated for studies of the relatively small auditory cortex, which is often convoluted, with high inter-individual anatomical heterogeneity (49). Overall, MEG and EEG provide complementary information with complementary SNR depending on the orientation and depth of the signal of interest and therefore it is beneficial to combine MEG and EEG where possible (45).

There are also practical differences between MEG and EEG. Because MEG is sensitive to magnetic fields, participants are required to be free from metal components that perturb the tiny signals from the brain, including belts or dental braces. Importantly for hearing research, participants with cochlear implants cannot be tested and hearing aids must be removed. Moreover, because recording devises are not attached to the scalp, head movement must be kept to a minimum as this can cause large discrepancies in source localization.

#### MEG recording devices

Modern MEG devices can be equipped with different sensor configurations, which have different sensitivities and noise-cancelation properties. These are magnetometers, planar or axial gradiometers, or a combination of these (50). A simple magnetometer is a single loop of wire, which detects the magnetic field perpendicular to the surface of the coil, and is sensitive not only to nearby signals but also those from distant sources. However, a magnetometer is also sensitive to unrelated noise in the environment. One way to suppress this noise is to use gradiometers (51), which consist of a compensation coil wound in opposite direction to the magnetometer and thus the homogenous part of the field is canceled out. As distant sources produce more homogenous fields than nearby sources, for a gradiometer sensitivity to nearby sources is greater (52). Therefore, the gradiometer behaves like a spatial high-pass filter that measures the gradient of the magnetic field instead of the magnetic field itself, accentuating signals from sources near the detection system, while minimizing the contribution from stronger distant sources. Higher order gradiometer devices can be obtained by increasing the number of opposite-wound coils. The axial gradiometer configuration consists of coils along the same radial axis, while planar gradiometers consist of coils that are in the same plane, providing different spatial derivatives of the magnetic field. For a detailed description of sensor types see Hansen et al. (53). The difference between sensor configurations become relevant in detecting deeper sources, which will be made clear later.

### Limitations

One needs to be aware not only of the capabilities of M/EEG, but more importantly of the limitations of these methods in order to produce results that are interpreted appropriately. The main drawback of M/EEG is the *non-uniqueness* of the inverse problem: the problem of estimating the sources of the electromagnetic signals from the fields and potentials recorded outside the head. But reconstructing the sources of the signals is not possible from the measured data alone and in general, the spatial resolution of these techniques is fundamentally underdetermined. This is because there are potentially an infinite number of sources that could have produced the same signal (54), while there are only a limited number of sampling points or recording devices. Moreover, multiple dipole sources can exist ~0.5 cm or less from each other, while EEG electrodes on the scalp are at a distance of ~1.5 cm from the cortical surface. MEG sensors are even further (roughly 3 cm). Therefore, if two dipoles with the same orientation and direction are relatively close to each other, compared to the distance at which they are measured, the resulting field will be indistinguishable from the field of a single dipole (55). The problem is more pronounced for sub-cortical sources. Hence, the inverse problem of M/EEG is said to be an *ill-posed* problem and estimating the precise sources within the brain is *non-unique*. Therefore, it is necessary to apply a set of realistic assumptions that renders the problem soluble (56). Source reconstruction techniques have evolved over the years and improved methods have become available to identify and localize the sources of oscillations within the brain, both in time and frequency, with ever improving spatial resolution. For a review of these techniques and their underlying assumptions, see Baillet et al. (56) and Michel et al. (57). Also, Hillebrand et al. (58) provide a detailed description of beamformers.

### The translation from animal to human studies

Animal studies formed the bulk of the early research in the neural mechanisms of tinnitus and have provided valuable information on changes that occur in various structures of the auditory system. Spikes and LFP oscillations recorded from animals may carry complementary information. Single- and multi-unit spiking activity reflect the output of a single, or a small number, of neurons, respectively. On the other hand, the LFPs mainly reflect localized processing and input to a neural population, recorded from the close proximity of the recording electrode (0.5–3 mm) (59–61). More precisely, LFPs reflect synchronized activity in a population of neurons, and consist to a large degree of the summed PSPs [for a discussion, see Logothetis et al. (62)]. Their amplitude (and hence that of M/EEG) is determined by the amount of current summation (60, 63) and conversely, they themselves can be predicted from multi-unit spiking activity (64), although this latter has been shown to be frequency specific (65).

**Figure 3:**
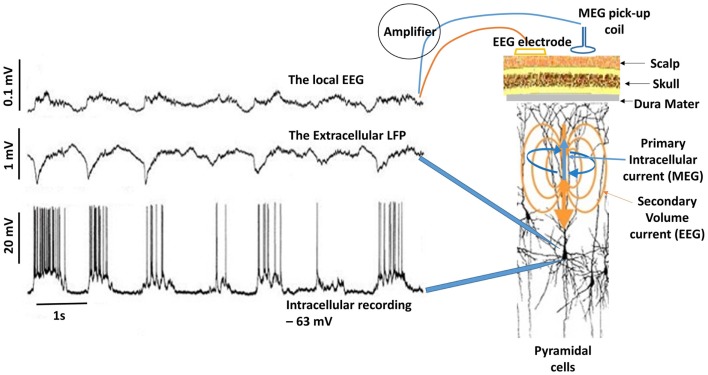
**Delta activity recorded from pyramidal neuron of a cat in the somatosensory cortex during deep sleep**. Bottom trace shows neuronal spiking due to depolarization in the membrane potential (action potentials) recorded at 1 mm depth. If synchronous enough, these can be recorded in the extracellular space as LFPs (middle trace). Top trace is the corresponding EEG, which is considerably diminished (about 10:1). In this case, the EEG was recorded by means of electrodes located on the surface and at a depth of ~0.6 mm. Notice the missing neuronal spiking after the third cycle in the bottom trace, which is reflected in the corresponding LFP and EEG [adapted from Contreras and Steriade (70)]. For simplicity, I have drawn the primary intracellular and volume currents (blue and yellow respectively), which are measured outside the head with appropriate sensors.

M/EEG oscillations have been shown to closely reflect the LFPs. In the visual cortex, the induced gamma (30–70 Hz) response to visual stimulation observed with intracranial LFP recordings in animals (59, 66) has also been observed in human beings using MEG (67). Hall et al. (68) showed that the MEG beamformer virtual electrode directly reflects the LFPs as recorded by Logothetis et al. (69) in their primate models. Figure 3 shows the relationship between neuronal spiking of a single cell, LFP recorded in the vicinity of the neuron and the corresponding intracranial EEG (iEEG) (70). It must be noted that the raw extracranial signals reflects synchrony rather than the local EEG as depicted in the upper trace. The recorded MEG signal is more likely to resemble this as the extracranial EEG is also distorted by the surrounding tissue.

Thus, M/EEG signals originate from the same neural activity that is recorded invasively from animals and they can be used reliably to assess tinnitus-related changes in human participants. Note that intracranial LFPs are weakly correlated with cognitive processes because of the partial information content provided due to the limited spatial sampling. In addition, intracranial recordings are typically made from non-human mammals and their interpretation in terms of processes in human beings is not straightforward. Therefore, despite methodological difficulties M/EEG are more appropriate techniques for investigating sensory, cognitive, and network processes in the human brain.

### Clinical applications of M/EEG

In addition to providing a research tool for the investigation of oscillatory neuronal responses, M/EEG are powerful tools that can be applied in clinical evaluation. Changes in oscillatory activity within different frequency bands can distinguish pathological from normal states. Generally speaking, focal slow-wave activity is characteristic of abnormal brain function and numerous M/EEG studies have reported changes in oscillatory activity in various conditions, including schizophrenia (71), Alzheimer’s disease (72), depression (73), tumors (74), migraine (75), and mild cognitive impairment (76). For schizophrenia in particular, numerous M/EEG studies have established abnormal oscillatory activity as a candidate mechanism for pervasive network impairment in this condition [see Uhlhaas and Singer (77) for a review].

The most notable clinical application of M/EEG is the diagnosis of epilepsy and the detection, and more recently localization, of inter-ictal epileptiform discharges (78). The traditional EEG is still the most reliable diagnostic tool for epilepsy and identification of epileptic spikes (79–81). The diagnostic yield of routine-EEG is estimated at 77.4% [Cascino et al. (82) and inter-ictal epileptiform activity can be identified in 92% of patients (79)]. More recently, MEG has proven even more successful in localization of epileptogenic zones prior to surgery, as well as the localization of functionally significant parts of the brain, or the eloquent cortex, in many patient groups, including those with tumors and lesional epilepsy (38, 39, 83, 84).

However, in contrast to epilepsy in which the aberrant neural activity manifests itself in spike and wave discharges, often recognizable in the recorded data with the naked eye, changes in neural activity associated with the tinnitus sensation are more subtle and undetectable with the naked eye in the recorded data. The interpretation of M/EEG oscillations in tinnitus requires careful analysis of the data, while taking into account changes in oscillatory activity due to factors unrelated to tinnitus and comorbid disorders such as depression, stress, hearing loss, and hyperacusis. While M/EEG are some way away from becoming a diagnostic tool for tinnitus, they are useful investigative tools for understanding neural oscillatory changes that correlate with the tinnitus abnormality.

### Types of M/EEG oscillations

The brain processes information by the activity of neural ensembles and their dynamic responses to rapid modulations of internal or external events. The strength of neural response reflects the sensitivity of neural receptive fields of different stimuli. One important feature of the brain is its ability to alternate between a synchronized and a desynchronized state, reflecting changes in cortical dynamics, that are initiated by external or internal events and represented, in the recorded signals, by high and low amplitude oscillations, respectively (35, 36, 85).

M/EEG data can be recorded in different ways, which reflect different brain processes and different generative mechanisms (86). In general, brain activity as recorded with MEEG can be classified as evoked, induced, or spontaneous. In short, spontaneous recorded activity refers to the ongoing brain activity in the absence of any stimulus, while both evoked and induced oscillations are related to external stimuli but differ in their phase relationship to the stimulus (87, 88).

#### The spontaneous activity

**Figure 4:**
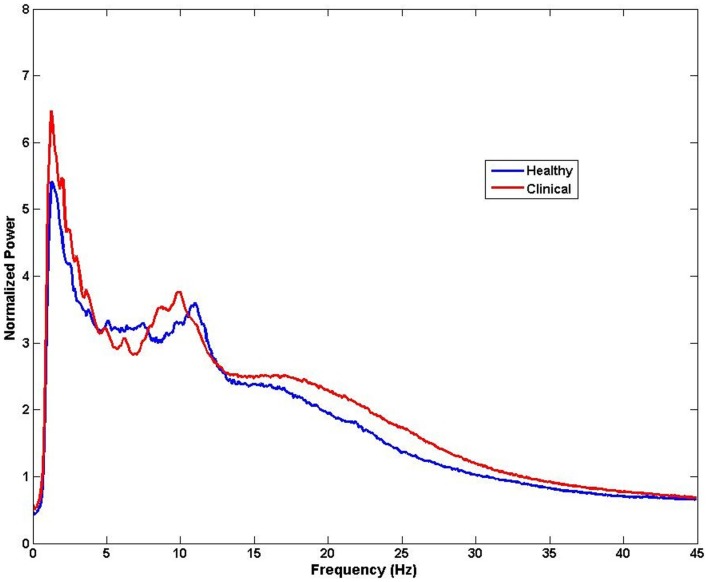
**Example of spectral analysis of MEG data for all sensors (*n* = 275)**. Differences in frequency between healthy and clinical samples can be obtained from activity measured at the sensors level for each participant and then averaged for each group. Note that the neural sources of these frequency effects cannot be speculated from this type of analysis.

The brain is active even in the absence of input from external stimuli. Spontaneous resting-state oscillations refer to the ongoing background activity when the brain is disengaged from performing specific tasks, and therefore they are not related to the presence of any stimulus. Therefore, changes in the resting-state magnitude and frequency of oscillations can be recorded with no reference to any events. Despite occurring spontaneously, these changes have been reported to be of behavioral significance (89). The ongoing brain activity can be analyzed by decomposing the distribution of signal intensity to obtain power and amplitude of the signal across frequencies. This analysis of frequency spectrum can be performed at sensor level, across all M/EEG sensors or a selection of channels corresponding to a region of interest, to obtain a coarse estimate of spectral content and subsequently compare between groups or variables of interest (Figure 4). However, it is important to note that this kind of analysis does not provide spatial specificity and the identifying the potential generators of these changes requires source analysis. Because, M/EEG signals are recorded in silence, evaluation of spontaneous recordings between tinnitus and non-tinnitus participants should reveal neural correlates of conscious tinnitus perception.

#### The evoked response

**Figure 5:**
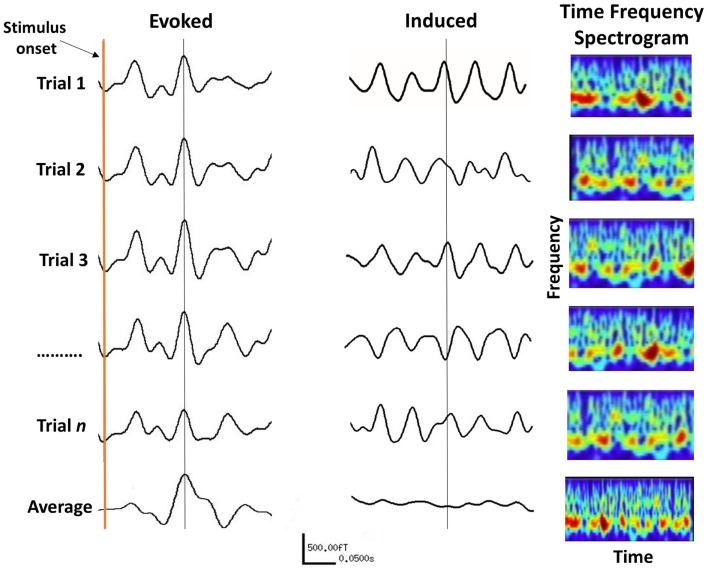
**The difference between evoked and induced M/EEG responses**. The AEF on the left is the linear average of many short trials whose responses are phase-locked to the onset of a stimulus. For induced responses (middle traces), linear averaging will remove the effect of interest due to variable phase relationship between trials. The information within induced responses is obtained by evaluating the frequency spectrum of each trial (right panels) over time, which is then averaged. The frequency information of individual trials is thus retained (bottom right panel).

Evoked responses appear after the onset of a stimulus and are phase- and time-locked to it, meaning that the signal of interest has a fixed time-delay to the stimulus. In general, event-related potentials are considered as the response of a stationary system to the external stimulus due to synchronous changes in afferent activity of neurons. Evoked power can be extracted by a simple linear method, such as the averaging of many trials to enhance signal-to-noise-ratio so that the noise is averaged out and the evoked response becomes apparent (Figure 5).

Auditory evoked potentials (AEPs) for EEG, and fields (AEFs) for MEG, are characterized by their amplitude and latency. Latency refers to the time taken from the onset of a stimulus, for the appearance of a response. The amplitude of the evoked response is believed to reflect the strength of the PSPs in a neural population, together with the amount of neural assemblies engaged in the processing of the stimuli (90). However, the amplitude of the averaged evoked responses also depends on the amplitude of the responses during the individual experimental trials and their phases across trials, which is called “phase-locking” (91).

Evoked responses are shown to have perceptual significance and be affected by cognitive factors (92). The most prominent component of the evoked response is the N1 (M100 or N1m for MEG) that occurs ~100 ms after the onset of the stimulus. The N1 response is the most widely used and robust response to assess auditory brain function, both clinically and for research. Sounds of different intensity and frequency are known to affect both latency and amplitude of the N1m (93, 94). Jacobson (92) argued that changes in the latency and amplitude of the N1 response can be used as objective indicators of neural abnormality in clinical groups, including cerebrovascular disease, schizophrenia, and tinnitus. The P2 (M200 or P2m for MEG) typically occurs ~200 ms post stimulus onset but its presence in normal participants is not ubiquitous with some investigators reporting P2m to be low in amplitude (95) or failing to report it all together (96). The next deflection of the evoked response is the P3 (M300 or P3m for MEG), which has a more variable latency and occurs between 250 and 400 ms post stimulus. The P3 is thought to reflect higher order brain function such as attention and other cognitive processes (97). The N and P refer to the negative and positive polarity of the waveform component from EEG recordings.

A related signal is the evoked steady-state response, which can be evoked in all sensory modalities by modulating the rate of a stimulus presentation. The “auditory steady-state response” (ASSR) is obtained either by clicks or by modulating the frequency or amplitude of a tone (98, 99). The neural responses are phase-locked to the stimulus modulation, or envelop of the stimulus in the case of complex auditory stimuli. Clinically, the ASSR is useful as a measure of hearing sensitivity, as it is less prone to confounds such as the state of consciousness or levels of arousal (100). The ASSR is typically amplitude modulated at 40 Hz at which responses are maximal. The 40 Hz ASSR can be localized in the primary auditory cortex in which the gradient of the tonotopic map depends on the carrier frequency with lower frequencies located more laterally and higher frequencies more medially (101, 102).

#### The induced response

Induced oscillations also occur after stimulation but are not phase-locked to the stimulus and the timing of their appearance varies between trials. Hence, averaging induced trials will remove the effect of interest due to their random phase relationship (see Figure 5). Instead non-linear analysis methods such as time-frequency decomposition must be applied to individual trials before averaging across trials (87). Induced oscillations can be defined as the modulation of the ongoing brain activity due to an internal or external event (or stimulus), which may last for the duration of that event. Changes in cortical oscillations reflect the response of neuron ensembles to the onset of events, such as the blocking of alpha activity on opening of the eyes [(103), cited in Pfurtscheller and Lopes da Silva (36)], and various cognitive functions (89). It has been shown that both the frequency as well as the amplitude of oscillations depend to a large extent on the size of the activated area (104). In principal, the faster, higher frequency brain rhythms originate from smaller cell assemblies with fewer neurons, compared to the slowly oscillating cell assemblies that emanate from a larger cortical area with a larger number of neurons. In general, there is an inverse relationship between the frequency and amplitude of induced oscillations, such that the amplitude of oscillations decreases with increasing frequency (36).

Spectral M/EEG responses are analyzed over traditional EEG frequency bands that exhibit different spatiotemporal characteristics. Typically, these are delta (1–4 Hz), theta (4–7 Hz), alpha (7–13 Hz), and beta (13–30 Hz), and the very high frequencies (>30 Hz) referred to as gamma activity. These can appear as either a decrease, or an increase, in cortical power, due to an increase, or a decrease, in synchronized activity. These phenomena are more commonly referred to as event-related synchronization (ERS) or event-related desynchronization (ERD) (36). ERS and ERD phenomena can occur across a whole range of frequencies.

The general notion is that evoked response paradigms are suited for studying bottom-up sensory processes, while induced responses reflect top-down higher order brain functions (87). The choice of which type of data to collect (spontaneous, evoked, or induced) will depend on the experimental question and the hypotheses being tested, which will in turn inform the choice of analysis method to employ.

### Source localization techniques

Given that the temporal resolution of M/EEG is in the order of milliseconds, traditionally, the analysis of data were limited to identifying the time course of brain activity. More recently, various methods have been developed, which not only provide the timing information but also a spatial estimate of the neural generators of the signals (58, 105). Therefore, time and frequency information can be obtained from improved spatially resolved loci rather than the coarse estimate relating to a particular lobe or hemisphere.

Localization of sources of neural activity from the measured M/EEG data requires solving the inverse problem. For localization of sources of M/EEG activity, an important methodological advance has been the implementation of the discrete source model, the dipole source analysis (54, 106). The equivalent current dipole (ECD) is a hypothetical source of the observed M/EEG fields and electrical potentials, which assumes that measurements are due to a single concentrated source. The evoked response reflects summed activity of different neuronal generators and dipoles reflect the location of the center of mass of all generators, biased toward the dominant contributor. Quadrupolar and higher order sources may be present, which tend to be weaker and thus masked by the presence of noise. The tested hypothesis is that an observed pattern of activity is statistically indistinguishable from that which would be produced by an ECD. A number of pre-determined *a priori* assumptions and constraints are necessary to limit the number and locations of active brain regions to those which make most physical sense. For example, auditory evoked fields may reasonably be presumed to arise from the auditory cortex and therefore the search area is limited to these regions. By manipulating the orientation, strength, and position of the hypothetical dipole to minimize the difference between the recorded and the modeled patterns, the optimal fit is found (107). A comprehensive account of the theoretical background of these models is given by Sarvas (46).

Dipole analysis is relevant in situations where there exists a clear averaged evoked response to time- and phase-locked stimuli in the recorded signals. Evoked response measurement does not take into account differences in the ongoing M/EEG oscillatory activity. Therefore, this method is less appropriate for tinnitus as only a limited portion of the brain can be studied, namely the sensory and motor cortices whose function are well synchronized to external stimuli. Oscillatory activity from sub-cortical structures that underlie cognitive functions, which are of interest in tinnitus, cannot be studied in this way because their activities are modulated by input from various regions of the brain and cannot be directly and precisely correlated with external events. Cognitive processes have much more variable latencies and averaging will cancel the activity of these sources (Figure 4).

Alternatively, distributed source models do not make any assumption on the location, number, and relevant timing of sources within the brain and provide 3-dimensional distributed source estimations, which can help to distinguish between several simultaneously active regions. These include “minimum-norm” solutions (105), which are based on a search for the solution with minimum power. Several variants of this approach have been proposed including standardized Low Resolution Electromagnetic Tomography (sLORETA) (108), which relies on the electrical current density estimate given by the minimum-norm solution and then “standardizes” it by its expected variance. However, minimum-norm solutions are underdetermined and diffuse, and favor solutions nearest to the sensors. Spatial filters techniques, such as beamformers (58, 109) are adaptive or data-driven approaches, which also contribute to noise-cancelation and provide time-frequency analysis of spatially resolved locations. Beamformers in particular have been found to reliably localize the sources of electromagnetic activity that match the localization ability of fMRI (110, 111).

Each source localization technique makes certain assumptions regarding the underlying sources of signals and has certain limitations. The choice of source localization technique must be based on clear experimental questions and the type of data acquired. Generally speaking, distributed source models are more useful for tinnitus given that tinnitus most likely involves activity of multiple brain regions, partly due to comorbidities, which still remain largely unknown. Michel et al. (57) provide a comparison of various analysis techniques with details of their strengths and weaknesses. Overall, distributed source models provide unprecedented means of investigating the underlying neural mechanism of tinnitus in human beings.

## Evaluation of Tinnitus Abnormality Based on the Evoked Response

A simple method for analysis of evoked responses is to measure the amplitude and latency of evoked responses and assess changes between different groups and/or variables of interest. Measuring the ratio of the N1/P2 response was established as an objective test of estimating hearing sensitivity in both children and adults (112, 113).

The first M/EEG study to assess changes in the evoked response in people with tinnitus was a MEG study performed by Hoke et al. (12). They measured the amplitude ratio of the recorded N1m/P2m complex to examine whether these are affected in participants with tinnitus. They demonstrated that the amplitude of the N1m response to a 1-kHz tone was significantly enhanced in participants with tinnitus compared to non-tinnitus controls, while the P2m was delayed, poorly developed, or missing. Consequently, the amplitude ratio of the P2m/N1m complex was smaller in tinnitus compared to controls. In a repeat study, the authors confirmed their earlier results and found that the P2m/N1m complex was smaller in tinnitus (114). A case report by Pantev et al. (115) of a tinnitus patient who suffered acoustic trauma, showed the gradual recovery of the N1m/p2m complex to normal levels over 6 months, which correlated with steady recovery from tinnitus. Using EEG, Noreña et al. (116) investigated the N1/P2 complex in response to a 1-kHz tone at different intensities (60–90 dB SPL). They found that patients with tinnitus had greater amplitudes and shorter latencies of N1 and P2 on the affected side at the highest sound level compared to controls, which they interpreted as possible increase in spontaneous activity due to reduced lateral inhibition.

Other studies contradicted the above findings. In an EEG study, Attias et al. (117) found a significant decrease in the amplitude of N1 and P2 for the TI group compared to controls, which is the opposite effect to the results of Hoke et al. (12, 114). Previously, MEG studies by Jacobson et al. (118) and Colding-Jørgensen et al. (119) had found no significant differences in amplitude and latency of N1m and P2m between TI patients and controls. Jacobson and McCaslin (120) found significantly smaller N1 amplitudes for 0.5 and 1 kHz tones in participants with tinnitus than normally hearing controls.

In these studies, the premise for using a 1 kHz tone to evoke a response is to examine general neuronal hyperexcitability in the auditory cortex. While a 1 kHz tone has been shown to optimally evoke N1 responses from the auditory cortex (94), the relevance of this stimulus to tinnitus is not clear. It is typically unrelated to the perceived tinnitus sensation and does not correspond to the edge frequency of hearing loss, and is thus inappropriate for assessing possible neuroplastic changes at the edge of hearing loss or in the deafferented frequency region of the cortex. Therefore, the use of 1 kHz tone seems inappropriate for addressing possible cortical changes in tinnitus, and has not been adequately justified by the authors.

Recently, we revisited the evoked response paradigm in tinnitus and examined changes in the N1m AEF amplitude to tones (121). Unlike previous studies that only used a 1 kHz tone, we used tones that were in some way related to the subjective tinnitus sensation or the associated hearing loss in each participant. These were: a tone corresponding to the individual audiometric edge frequency, a tone corresponding to the dominant tinnitus pitch, a tone within the area of hearing loss, and a “control” tone in the region of normal hearing. To distinguish between the effect of reorganization due to tinnitus and that due to hearing loss, two control groups were recruited; one with clinically normal hearing and no tinnitus and another with matched hearing loss but without tinnitus. We found no differences in the amplitude of the N1m between tinnitus and the control groups. In the tinnitus group, the N1m amplitude for the dominant tinnitus pitch was significantly smaller than for the control and audiometric edge frequencies and was similar to the tone within the hearing-loss region. Within each group, the N1m amplitudes for the tone at the edge frequency were not significantly different to the control tone. There was no difference between the N1m amplitude for the tinnitus pitch and hearing-loss frequency. We concluded that the N1m is not an adequate measure of tinnitus-related cortical reorganization of frequency coding (121).

Thus given the disparity between the results, the early findings based on N1 or N1-P2 complex are disputable on various grounds. First, the P2 is not a ubiquitous presence in the evoked response, and its amplitude varies between people independent of clinical factors, making the assessment of the P2 response an unreliable indicator of central abnormality in tinnitus. The N1-P2 complex has been shown to be susceptible to subject drowsiness (97) and its use as a viable test in the clinical setting has been dismissed (122). Second, not all studies report the same changes in the evoked N1/P2 response. Third, these findings are based on sound evoked responses that emanate from the auditory cortex, implying that the tinnitus abnormality is primarily related to the tinnitus sensation and directly due to aberrant neural activity within the auditory cortex. Recent neuroimaging studies indicate a less direct role for the central auditory system in tinnitus and a more significant role for non-auditory parts of the brain, such as the limbic system.

### Source localization in tinnitus based on the evoked response

Identifying the cortical generators of N1/P2 responses is also imprecise, due in part to the inverse problem described earlier. In addition, an auditory stimulus activates multiple neural populations, which then summate to produce multiple waveforms and complex spatiotemporal fields and topographies on the scalp. Therefore, the evoked response represents both summation and cancelation of neural activity from the active cortical regions. Näätänen and Picton (94) suggest that N1/P2 consist of overlapping subcomponents generated by primary as well as secondary auditory cortices. A study by Scherg and Von Cramon (106) using dipole analysis, found results that suggested the tangential source could reflect the activity of Heschl’s gyrus while the radial source could reflect the activity of the superior temporal gyrus (STG). Using distributed source models applied to intracranial recordings, Yvert et al. (123) showed that N1 activity is more distributed, spanning the Heschl’s gyrus and sulcus, Planum Temporale, and the STG. Other studies suggest that the Heschl’s gyrus is more easily detectable by EEG than MEG, which may be due to increased activity of radial sources (which are located in the cortical gyri) undetected by MEG (124).

In a MEG study, Mühlnickel et al. (125) reported changes in the tonotopic map of four patients with tinnitus using dipole analysis to localize the sources of N1m responses. They showed that in participants with tinnitus, the dipole location to the response evoked by a tone similar to the tinnitus pitch was shifted compared to the linear arrangement observed in non-tinnitus controls. However, these results have not been replicated since by any other group and require cautious interpretation. The localization of the tonotopic map in healthy participants based on the evoked M/EEG response is notoriously difficult with many contradictory results [see Lütkenhöner et al. (126) for a mini review of these studies and a more in-depth discussion of this issue]. Previously, researchers have urged cautious reservation when interpreting dipole locations derived from the evoked response (127, 128). The problem is made even more complex amid high inter-individual variability (126, 129–131). Mühlnickel and colleagues used only four tinnitus participants and thus the results could easily be due to individual variability. Therefore, while the study by Mühlnickel et al. (125) is often cited in support of the tonotopic reorganization model of tinnitus, the reported change in dipole locations do not necessarily reflect an effect of plasticity, nor of tinnitus. Other problems with the interpretation of this study were described in Adjamian et al. (13).

Dietrich et al. (132) used MEG to examine auditory cortex expansion for frequencies at the edge of hearing loss based on dipole strength values in eight participants with tinnitus. Three tone frequencies were used comprising of one corresponding to the lesion edge frequency, and two in the area of normal hearing. They found a significant increase of dipole moment value for the lesion edge frequency compared to the frequencies in the normal hearing region, which they conclude is due to the expansion of the cortical representation for the lesion edge frequency. However, the strength of dipole moment can be determined not only by an increase in the number of activated neurons but also by an increase in neural synchrony due to increased synaptic transmission in the existing neurons. M/EEG amplitudes do not distinguish between these neuronal events. Therefore, it is equally likely that the enhanced dipole strength observed by Dietrich and colleagues is due to increased synchrony within the existing neurons at the lesion edge without an expansion of this area. Moreover, the authors do not provide information about the pitch of the tinnitus percept in their participants, and thus it is not possible to know whether the perceived sound corresponded to the expanded area at the edge of the hearing loss. Numerous studies show that for most people with tinnitus the dominant tinnitus pitch either falls within the area of hearing loss (133, 134) or spans a broad spectrum within the area of hearing loss (135–138). Therefore, the implication of the findings by Dietrich et al. (132) for a central processing mechanism of tinnitus is unclear.

Overall, the ECD approach is insufficient to expose the neural underpinning of the tinnitus abnormality. The underlying assumption of this model is that the source distribution of a particular waveform is a single dipole located in a specific area of cortex. It does not account for sources of global oscillatory phenomena, or the interaction between neural ensembles and thalamic input from sensory stimuli. For these reasons, it cannot account for the involvement of other brain regions that maybe simultaneously active, such as those involved in top-down processes.

### Evaluation of tinnitus abnormality based on the ASSR

Diesch et al. (139) were the first to investigate tinnitus with ASSR using 40 Hz modulated tones of various carrier frequencies. They found a correlation between the subjective rating of tinnitus intrusiveness and the amplitude of the ASSR response with carrier frequencies matched to the tinnitus pitch showing the most enhancement. However, no control group was used for comparison and because the degree of hearing loss was not accounted for in the correlation analysis, a contribution from the severity of hearing loss could not be ruled out. In subsequent studies, Diesch et al. (140, 141) factored out the effects of hearing thresholds and age by closely matching their tinnitus and non-tinnitus controls and confirmed the relationship between neural activity underlying the ASSR and tinnitus. Wienbruch et al. (142) used the 40 Hz ASSR using different carrier frequencies (384–6561 Hz) to compare tonotopic frequency representations between participants with tinnitus and normally hearing controls. They found that in participants with tinnitus the ASSR frequency gradients were shifted bilaterally. They suggested that this altered frequency representation in tinnitus may reflect reduced inhibition in deafferented regions of the primary auditory cortex. Moreover, the strength of dipole moments was also increased in tinnitus suggesting elevated response from synchronized neurons to the 40 Hz modulated stimulus. However, in this study, tinnitus and non-tinnitus controls were not matched on hearing threshold and therefore it is not possible to determine whether the reported effect is due to hearing loss or to tinnitus.

## Spontaneous and Induced M/EEG Responses in Tinnitus

Frequency is an important feature when differentiating between normal rhythms and the pathologically abnormal activity. Therefore, when reporting changes in induced or spontaneous oscillatory activity in tinnitus, it is necessary to specify the frequency band in which the changes are observed. An “increase” in oscillatory activity within a certain frequency band is only meaningful if the rhythmicity was not present as a spectral peak in a baseline measurement. Similarly, a “decrease” in oscillatory power has meaning only if the change in rhythmic activity was present as a clear peak in the power spectra of a relevant baseline (143). This presents an obvious problem for tinnitus given that it is an ongoing sensation and hence obtaining a baseline measurement (where tinnitus is absent) in the same participant is not usually possible. For this reason, many investigators examine the resting-state spontaneous M/EEG in people with tinnitus, and compare the frequency spectrum of the data to those from participants without tinnitus.

Numerous studies have assessed spontaneous brain oscillations in tinnitus compared to controls with somewhat mixed results (144–149). While the MEG studies by Weisz and colleagues found increase in the slow-wave delta activity in tinnitus participants compared to controls, the EEG study by Ashton et al. (144) failed to report any differences in the low frequencies. Using MEG, Weisz et al. (145) also reported decreased alpha activity from the same regions as increase delta activity which they attributed to a disinhibition of the ‘normal’ brain rhythms due to hearing loss, concomitant reduction of inhibitory neural activity, and increased excitatory drive.

Abnormal focal slow-wave (delta and theta) activity appears to be related to a number of pathophysiological conditions (150) including tumors (151), stroke (152), depression (153), Schizophrenia (154), and Alzheimer’s disease (155). The TCD model by proposed by Llinás et al. (18, 156) predicts that tinnitus is associated with enhanced activity in low frequencies (delta and theta) due to reduced lateral inhibition, which disinhibits gamma oscillations in the neighboring cortex. Accordingly, abnormal gamma oscillations in tinnitus are not due to increased spontaneous activity but an “edge effect” in neurons surrounding the theta-locked areas of the auditory cortex. Weisz et al. (146) reported increased gamma activity in the contralateral auditory cortex of tinnitus participants. Ashton et al. (144) also reported increased gamma activity but this did not track the laterality of the tinnitus percept. van der Loo et al. (148) found that the increase in gamma activity is dependent on the subject intensity of the tinnitus percept. In an EEG study, Balkenhol et al. (157) report increase in gamma (31–64 Hz) as well as delta (0.5–3 Hz) activity with increasing tinnitus loudness in the frontal regions contralateral to the tinnitus side. However, for both studies, no analysis to estimate the sources of these changes was performed. Nevertheless, these studies showed that the analysis of spontaneous M/EEG activity can reveal significant information about the neural changes in tinnitus.

The MEG study by Ortmann et al. (158) is perhaps the nearest example of obtaining induced responses in tinnitus. They examined a group of musicians without chronic tinnitus but who reported transient tinnitus immediately after band practice. The musicians’ hearing and tinnitus levels were examined before and after exposure to loud music. Temporary tinnitus and hearing loss were detected in both ears, which accompanied increased gamma activity in the right auditory cortex of most participants.

Finally in this section, it is imperative to mention a significant concern relating to the interpretation of induced gamma response recorded with EEG. Yuval-Greenberg et al. (159) observed that with channels referenced to the nose, as is common practice in EEG, the observed transient broadband “gamma oscillations” (30–90 Hz) are generated in occipital electrodes around 300 ms after stimulus onset, and are time-locked to the onset of involuntary eye movements, or saccades. Therefore, the most likely source of this gamma activity is ocular contamination of the EEG reference rather than neuronal oscillations. The results of this study provide serious concerns with EEG and the validity of observed gamma oscillations (160), at least as far as the visual system is concerned. Whether auditory gamma activity observed with EEG is compromised by similar issues is not known and requires investigation.

### Residual inhibition and masking

Two strategies have been employed to suppress the tinnitus percept and examine changes in oscillatory activity. These include residual inhibition (RI) and masking. Both RI and masking are behavioral measures that can partially or completely suppress the tinnitus sensation for a short period of time. By modulating the intensity of the tinnitus percept, researchers can quantitatively assess changes in brain dynamics when tinnitus is heard and when it is partially or completely suppressed. The physiological effects of noise masking and RI are different. While masking drowns the tinnitus sound while it is being heard, RI is a temporary, partial, or complete, suppression of tinnitus after the noise has been removed (136, 137). RI can last up to a few minutes and therefore it is possible to use a typical box-car experimental design to evaluate changes in oscillatory activity when tinnitus is heard and when it is absent.

Using MEG, Kahlbrock and Weisz (161) examined changes in spontaneous brain activity during RI and periods of tinnitus. They found a significant reduction of power in the delta frequency band during RI in the temporal region but the alpha or gamma frequency bands were not altered. Also using MEG, Sedley et al. (162) aimed to examine changes in oscillatory activity brought about by RI in participants with chronic tinnitus. While tinnitus was at least partially removed in most participants (*n* = 14), in a few others it became worse (*n* = 4) after the masking sound was removed, which they termed residual excitation (RE). Interestingly, the observed RE was accompanied by bilateral reduction in gamma power in the auditory cortex, despite the increase in the perceived tinnitus intensity. One participant’s reduction in tinnitus during RI was accompanied by increase in auditory cortex gamma oscillations. Moreover, there were no changes in delta/theta power in the auditory cortex or elsewhere in the brain. Taken together, although correlational, the results seem to oppose the TCD model, as they imply that gamma oscillations play a role in reducing the tinnitus percept.

In a recent MEG study, we took a different approach to examining the predictions of the TCD model (149). Using a white noise to mask the tinnitus percept, we employed a beamformer virtual electrode analysis technique to examine oscillatory changes in the auditory cortex of tinnitus participants with and without normal hearing based on its clinical definition (thresholds ≤20 dB between 250 and 8 kHz). At the group level, we found significant increase in slow-wave delta band (1–4 Hz) activity in both groups of tinnitus participants, with and without hearing loss compared to normal hearing controls and those with hearing loss alone. More importantly, we found a concomitant decrease in the delta activity during masking in those participants who experienced inhibition of their tinnitus but not in those who did not experience this inhibition. In other frequency bands, we did not find a significant difference at the group level between the groups. Although a few of our tinnitus participants did have significantly increased gamma activity, this was not significant at the group level. The results only partly supported the TCD model. We concluded that the slow-wave delta activity is a signature of tinnitus abnormality in the auditory cortex and appears to be unrelated to the degree of hearing loss, at least as far as the clinical definition of hearing loss is concerned (149). The results also indicated that the MEG signal is a reliable indicator of changes in the tinnitus percept in the auditory cortex and virtual electrode analysis can provide spatial specificity of these changes.

Broadly speaking, masking and RI are believed to suppress the abnormal hyperactivity that is thought to give rise to the tinnitus sensation (137). However, Roberts et al. (15) found that the ASSR amplitude is increased following masking in tinnitus patients compared to the pre-masking amplitude but not in normally hearing controls. This finding counters the idea that masking habituates the neuronal spontaneous firing. While masking and RI can silence the tinnitus percept, they only provide an incomplete picture of the tinnitus abnormality in the brain.

It must also be noted that currently, M/EEG analysis techniques (and fMRI for that matter) do not provide the spatial resolution required to disambiguate the cortical sources of slow wave and gamma activity from one another in adjacent regions of the auditory cortex. This problem is further complicated by the existence of multiple frequency gradients in the auditory cortex (163, 164). Thus the localization of the sources of gamma and delta/theta activity at the frequency edge of hearing loss, in accordance with the predictions of the TCD model is a major challenge. The model remains a conceptual framework to further explore tinnitus-related temporal abnormalities in the brain.

### The role of non-auditory brain regions in tinnitus

In general, information processing within the brain is thought to be organized such that any two, or multiple, areas are connected by reciprocal connections. Because external information arrives in the brain via various sensory modalities, the sensory end of the reciprocal connection in the brain is taken to be the starting point of this process (87). This feed-forward hierarchy is often referred to as “bottom-up” processing, going from lower level sensory stage to higher order, emotional, attentional, and cognitive brain functions. Conversely, the process can begin at the higher end of this hierarchy with functions, which are simultaneously active as the sensory information arrives. This kind of feedback information flow is referred to as top-down processing, involving activity from the sub-cortical structures of the limbic system and the frontal lobes (87).

More than 20 years ago, Jastreboff (165) suggested that tinnitus is a top-down process and that neural changes underlying the tinnitus abnormality extends beyond the auditory cortex. Numerous human neuroimaging studies provide support for this stance, indicating that the neural changes underpinning tinnitus are not confined to the auditory system and that non-auditory, higher order functions such as attention, memory, and emotion are also affected. Therefore, tinnitus and the associated psychological and emotional distress are thought to involve top-down processing involving multiple parallel and partially overlapping networks, each with a different pattern of oscillatory activity. Current debate has thus moved on to which non-auditory structures are involved, and whether the observed oscillatory changes in these structures are directly related to the emergence of tinnitus, or whether they are symptoms of the abnormal neural activity in the auditory system. de Ridder et al. (24) suggest that for tinnitus perception to be consciously perceived, it is required that sensory input to the auditory cortex is functionally integrated with a network of frontal and parietal areas, and the posterior insula, similar to the sensation of pain. This is consistent with the aversive emotional state experienced by many tinnitus sufferers and involves abnormal input from structures of the limbic system, including the amygdala. Human lesion studies have shown that the amygdala is an important part of the neural circuitry critical for cognitive evaluation of emotional content of sensory stimuli (166, 167). Based on mainly the analysis of anatomical MRIs, the gating mechanism proposed by Rauschecker et al. (19) implicates sub-cortical structures including the amygdala, the nucleus accumbens, dorsal raphe nucleus, and ventromedial pre-frontal cortex. However, M/EEG evidence for the involvement of these regions is scant while those from structural studies are not convincing [see Adjamian et al. (23) for a review].

Using EEG, Balkenhol et al. (157) reported a significant correlation between tinnitus-related distress and delta band (0.5–3 Hz) power and that high-distress was associated with significantly higher theta band (4–7 Hz) power compared to low-distress tinnitus. No localization of the sources of the observed signals was reported. Also using EEG, Joos et al. (168) distinguished between the neural circuits underlying the transient state of distress and the constant state of depression in tinnitus patients. They found a positive correlation between tinnitus distress and the frontopolar, orbitofrontal, and postgenual and subgenual anterior cingulate cortex in the low to middle range beta band (13–21 Hz). For depression, they found a positive correlation between depression and alpha activity in the frontopolar and orbitofrontal cortex, and upper beta range (22–30 Hz) activity in the subgenual anterior cingulate cortex. Depressive feelings exclusively correlated with the left frontopolar and orbitofrontal cortex in the upper alpha band (10–12 Hz). Moreover, higher patient scores on depressive feelings were correlated with more activity in the left orbitofrontal cortex for this upper alpha band. The overall conclusion from this study is that tinnitus-related depression and distress are associated with specific changes in brain activity of separate neural pathways, and that both emotional aspects have specific neural circuit embedded within a larger common network. However, the authors used sLORETA as means of identifying the sources of the observed signals, which is a coarse measure of source localization with notoriously low spatial resolution (57) and does not differentiate between adjacent sources. Moreover, sLORETA localization accuracy is susceptible to even small amounts of biological or environmental noise in the data (169). sLORETA localization error is minimal for single dipolar sources but increases when multiple source are active simultaneously. Consequently, the probability of localization error is high as two simultaneously active nearby sources can be misleadingly represented as a single source. Realistically, tinnitus-related stress and depression require the activation of multiple adjacent areas and therefore the reconstructed sources from studies that employ LORETA must be cautiously evaluated in the context of these limitations. Finally, it has been claimed that the underlying assumptions of the LORETA inverse solution are erroneous (170). It is not my intention here to enter a debate on technical issues relating to various source reconstruction techniques but rather to stress that all presented M/EEG results require cautious interpretation in the light of technical limitations of the analysis techniques used. Likewise, the results by Joos et al. (168) require validation and confirmation, not only from other research groups but also using other source imaging techniques.

### Detection of deep sub-cortical sources with M/EEG

Due to the deep position of structures involved in top-down processing within the brain, the question for M/EEG is one of their sensitivity to detecting sub-cortical neural sources. Numerous factors contribute to the detection of a signal deep in the brain, including the number of active neurons (area) or synapses; the orientation of the electromagnetic field to recording electrodes; signal to noise ratio; and the magnitude of the signal generators and their distance from the electrodes. Evoked responses have larger SNR compared to spontaneous responses due to repeated stimulation and averaging of the signal. But as discussed earlier, tinnitus is likely to involve top-down processing in which oscillatory events occur with variable time courses and phases and spontaneous rather than evoked response paradigms are more suitable for its detection. For example, the amygdala is located deep in the anterior medial temporal lobe, meaning that the signal generated by it is significantly attenuated on the scalp, particularly for spontaneous recorded activity but even for evoked responses that typically have higher amplitudes. This means that the detection and localization of deep sources is a challenge for M/EEG.

For EEG, deep dipole layers (for instance, in the thalamus) make much smaller contributions to scalp potentials due to the greater distance between sources and electrodes (34). For example, the thalamus has a closed dipole field due to its round structure (17), which decreases the likelihood of detecting its related electromagnetic activity directly. In general, localized generators are not a feature of EEG data as signals related to cognitive processes involve distributed cortical tissue and separate brain regions (33). With MEG, signal strength diminishes rapidly with increasing distance from the current generating source. Overall, MEG sensitivity to deeper sources is inferior to EEG and depends on the configuration of the magnetic sensor that the scanner is equipped with. Magnetometers are more sensitive than first order gradiometers, which are in turn more sensitive than second order gradiometers and so forth (171). Planar gradiometers are least sensitive to more distant signals and are optimally tuned to superficial cortical sources that are directly beneath them (172).

**Figure 6:**
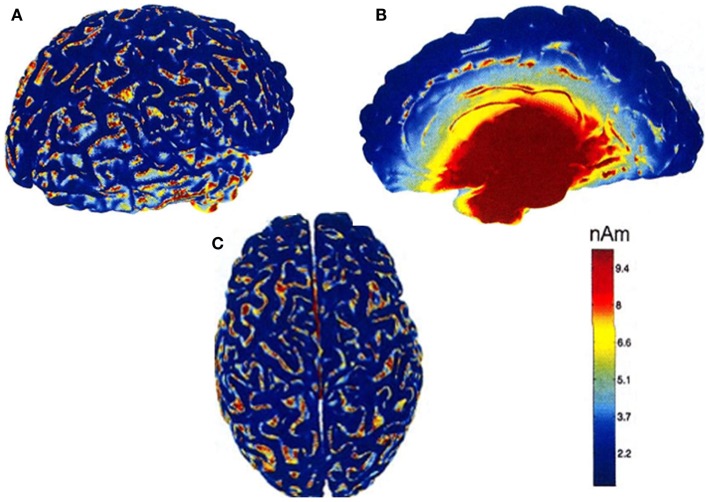
**Maps of the source strength that is needed to obtain a detection probability of 0.7 in one participant [from Hillebrand and Barnes (47)]**. Data were recorded with a 151 MEG scanner with third order gradiometer configuration. The folded cortical surface is viewed from the right **(A)**, left-midline **(B)**, and top **(C)**. The colormap of the source strength is clipped at 10 nAm. For a detection probability of 70% reasonable source strength is sufficient to detect signals from most areas of the brain. For deep sources, a source strength of at least 10 nAm is needed in order to obtain a high detection probability. Figure adapted from Hillebrand and Barnes (47).

Based on the estimation by Hillebrand and Barnes (47), for MEG the probability of detecting a source as deep as the amygdala can be as low as 10% (Figure 2). Hillebrand and Barnes (47) also estimated the strength of magnetic field required to detect sources with a probability of 70% for all regions of the brain (see Figure 6). From this, it can be seen that recording from sub-cortical structures requires stronger responses compared to cortical sources by at least four orders of magnitude. More recently, Goldenholz et al. (45) confirmed this assertion and also found that the SNR of EEG was low in the inferior frontal areas, possibly due to inadequate sampling of the scalp potential distribution with the low electrode array they used (*n* = 70).

Studies that report successfully recording signals from deep sources are rare. Using MEG, Tesche (173) identified magnetic waveforms for thalamic components of evoked responses to median nerve stimulation and localized the activity to the thalamic region using a restricted dipole analysis technique. But the spatial resolution of the thalamic activity was in the order or centimeters. In another study, Tesche (174) reported detection of ongoing hippocampal oscillatory 4–12 Hz activity. Also using MEG, Tesche and Karhu (175) identified theta activity emanating from the hippocampus using a memory paradigm and an elaborate signal analysis algorithm using dipole analysis and signal source projection filtered in the theta band. A similar study by Cornwell et al. (176) also recorded theta activity from human hippocampal and parahippocampal cortices using a beamformer analysis technique. Spatial filtering techniques, such as beamformers, can be used to reconstruct time course of oscillatory activity from any part of the brain, including deep sources, provided that we have sufficient *a priori* knowledge regarding the sources of the activity.

### Detection of sub-cortical structures in tinnitus

Vanneste et al. (177) recorded spontaneous EEG to examine tinnitus-related distress in participants with high, medium, and low distress. They found increased alpha activity in tinnitus participants with high, compared to low, distress in emotion-related areas, including anterior cingulate cortex, the insula, parahippocampal area, and amygdala. In their EEG study of tinnitus-related distress and depression, Joos et al. (168) demonstrated a significant positive effect between levels of distress and the upper beta band (21.5–30 Hz) in the parahippocampal area, which is located deep below the subcallosal region. However, both studies used sLORETA to estimate the sources of recorded signals, which produce a blurred image of source activity. As described in the preceding section, sLORETA is prone to localization error in the presence of adjacent sources and has a relatively low spatial resolution with the result resembling a distributed rather than a point source (57). This is also evident by the image provided in the paper by these authors themselves. Thus these results also require confirmation from similar studies by other groups as well as different source localization techniques.

Whether it is possible to record and accurately localize spontaneous activity directly from the amygdala and other sub-cortical structures is debatable and remains a serious challenge for most researchers and the signals from these structures are relatively poor. Given that the amygdala is organized in nuclei resembling a closed field structure, it is unlikely to generate a sufficiently measurable electromagnetic signal on the scalp. This does not mean that functions, which involve the amygdala do not generate scalp signals, but highlights the fact that deep sub-cortical sources with low SNR cannot be considered as accurately localized as cortical sources with high SNR. There are numerous connections that facilitate the interaction between the amygdala and other brain regions, such as the pre-frontal cortex, whose activity can be measured directly by M/EEG.

## Measures of Brain Connectivity

Generally speaking, neural activity underlying sensory processes are thought to be modulated by top-down mechanisms that integrate information from neural assemblies in different modalities depending on the context, experiences, and prior knowledge (89). Dehaene et al. (178) have suggested the theory of “global workspace,” according to which activity in the sensory areas only become conscious when they are functionally connected with a large fronto-parietal network. But the specific mechanism of this neural interaction remains a topic of some debate and the question of how different brain regions in large-scale networks communicate with each other is of increasing interest in neuroimaging research.

**Figure 7:**
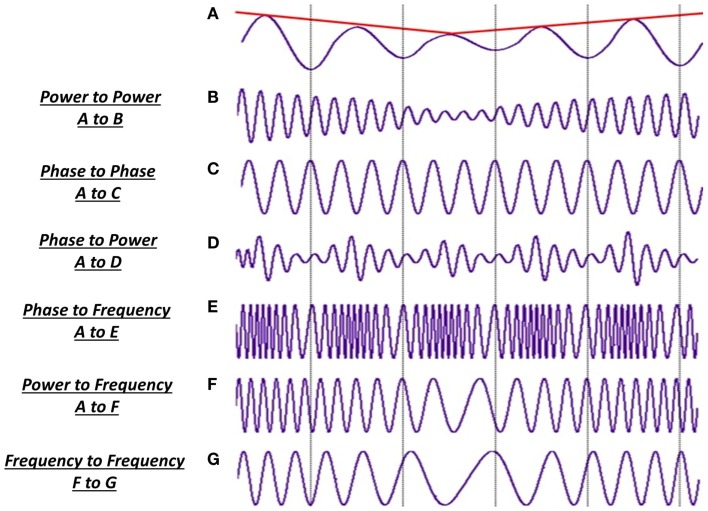
**Different types of cross-frequency coupling**. The interaction between brain regions can be assessed by measuring transient synchronization between the recorded activities. **(A)** Slow-wave activity in the theta band (8 Hz) with fluctuation power (red line) but stable frequency. Gamma frequency oscillation (*B-G*) can interact with this signal in the following ways: **(B)** amplitude in the gamma oscillation can correlate with that of the theta band irrespective of changes in phase of the two signal; **(C)** phase-locking between two signals occurs as one oscillation period of signal *A* corresponds to three periods of signal *C*, which remains locked or fixed; **(D)** modulations in the amplitude of the gamma oscillation are correlated to the phase of the slow-wave activity; **(E)** modulations in the frequency of gamma oscillation is correlated with the phase of *A*; **(F)** frequency modulations in the fast gamma activity is coupled to the amplitude of modulations in the slow-wave theta activity; **(G)** changes in one frequency range are induced by changes in another frequency range. Adapted from Jirsa and Müller (181).

The most likely mechanism to facilitate functional connectivity between different regions is thought to be transient synchronization of neuronal oscillatory activity, which binds the activity from distributed neural ensembles into a coherent representation of cognitive and sensory functions (87). Perceptual binding and functional integration require large-scale neural synchrony and coordinated activity of distributed neural ensembles (179). The scope of this large-scale synchronization is neural assemblies which are >1 cm apart (87), such as, for example, assemblies across hemispheres or between auditory and pre-frontal cortices. Transient synchronization can be measured by various forms of coupling, using frequency, phase, and amplitude of the signal. Jensen and Colgin (180) and Jirsa and Müller (181) summarize the different principles of cross-frequency interdependencies between signals (see Figure 7), which include power to power, phase to phase, phase to frequency, and phase to power.

### Functional vs. effective connectivity

When assessing the interaction between distinct brain regions, a key distinction is between functional and effective connectivity (182). Functional connectivity is mainly a statistical concept, which measures the level of dependence between distributed, and spatially distinct neural signals. It assesses the interaction between different cortical regions by evaluating the cross-correlation of the signals between different sensor sites without specifying the direction of this interaction. Effective connectivity, on the other hand, attempts to establish, which system influences the other (183). This depends on first establishing functional connectivity and then the use of a hypothesized model. Friston (184) provides a comprehensive review and discussion of these concepts from the perspective of neuroimaging techniques.

### Brain connectivity in tinnitus

As discussed, it is now generally agreed that tinnitus is a disorder involving a distributed network of auditory and non-auditory structures. The appearance of tinnitus and its associated emotional psychological effects, such as depression, anxiety, and sleep deprivation critically depends on functional integration between areas that subserve these functions and auditory cortical regions. The analysis of functional connectivity can reveal a “tinnitus network,” providing at least a putative link between various brain structures that are involved in tinnitus and the associated perceptual, psychological, and cognitive factors. Given that tinnitus is now believed to involve a network of sensory and non-sensory brain regions, few studies have emerged showing that normal communication between disparate brain regions is altered in tinnitus. The first study to assess brain connectivity in search of a “tinnitus network” was carried out by Schlee et al. (185). They found phase synchrony between the anterior cingulum, the right frontal lobe, and the right parietal lobe, which correlated with the person’s subjective rating of tinnitus distress. However, because they used a source montage rather than precise source analysis, they could not infer an interpretation of precise location of synchronized sources. Moreover, as they did not attempt to segregate tinnitus from hearing loss, their phase synchrony effects could be due to hearing loss rather than tinnitus saliency. In a follow-up study, Schlee et al. (186) found that phase synchrony in the alpha and gamma bands were negatively correlated across subjects. They also showed that the gamma network changed with the duration of tinnitus. In tinnitus that had existed longer than 4 years, the gamma network was more widely distributed and included frontal and parietal regions while in tinnitus of less than 4 years, gamma network in the left temporal cortex was predominant. In yet another study of effective connectivity to assess the direction of change, Schlee et al. (187) mapped cortical network hubs. The found differences on group level between tinnitus and controls in the gamma frequency range between pre-frontal cortex, the orbitofrontal cortex, and parieto-occipital regions whose connection strength was most strongly affected by tinnitus. Recently, Schlee et al. (188) have proposed the Global Brain Model (GBM), which suggests that the interplay between a sensory and a global component results in the tinnitus sound. The sensory component comprises the auditory cortices, which produces gamma oscillations. Similar to the TCD model, these gamma oscillations arise from decreased alpha activity caused by deafferentation, which leads to disinhibition and an edge effect. The global component consists of distributed and interconnected brain areas including fronto-parieto-cingulate network, which amplifies the auditory neural activity by top-down influence.

These early results are encouraging as they indicate that tinnitus is related to network changes involving both auditory and non-auditory regions and appropriate the “global workspace model.” The results also emphasize the potential of M/EEG in examining the tinnitus abnormality at the level of brain networks. Similar to the TCD model, the GBM model too makes a number of specific predictions that can be tested in human beings using M/EEG. Overall, the interaction and functional relationships between different brain regions in tinnitus requires much further research based on clearly defined hypotheses. However, it is worth noting that the relationship between regions based on large-scale integration is only correlative. Despite the early result by Schlee et al. (185) more evidence that changes in synchronous activity can affect tinnitus perception remains to be established by future research.

### Confines of connectivity measures with M/EEG data

A major interpretative problem for brain connectivity based on neural synchronization analysis is that due to volume conduction of brain tissue, the electrical activity recorded at a scalp site does not represent just the local neural activity directly below the recording device (183). Fields generated by a single source are present at different sensors, meaning that cross-correlations do not necessarily represent functional connectivity between independent neural sources. In such cases, the apparent connectivity between the sources is completely spurious. This problem of field spread has serious implications for interpretation of results and necessitates that connectivity analysis is performed in source space. In addition, source space analysis allows direct indication of anatomical location of the interacting brain regions. Schoffelen and Gross (183) provide a detailed description of this and other methodological problems associated with connectivity analysis. Improved analysis techniques have become available, which allow connectivity analysis in source space with unprecedented spatial resolution (189, 190). Furthermore, different investigators may be assessing different aspects of interegional interaction and one experimenter claiming to evaluate functional (or effective) connectivity does not mean he/she assesses the same quantities as another researcher. Therefore, comparisons of functional and effective connectivity from different studies should be done with caution as neither measure is a single concept (191).

## Possible Sources of Variability between M/EEG Studies of Tinnitus

M/EEG data are extremely rich in temporal information they convey, which are sensitive to both external and internal events. One has to be certain that any observed differences between tinnitus and control populations are not due to random variations between participants, or non-tinnitus factors that may change brain oscillations. Considering the subtlety of the tinnitus sensation, the changes in M/EEG rhythms are also likely to be very subtle. Given the often small sample sizes in M/EEG studies of tinnitus, the conflicting results suggest that the effect sizes and statistical power will also be small. This problem is highlighted with an absence of strong *a priori* information and hypotheses regarding the spatiotemporal changes in tinnitus.

Random inter- and intra-individual variability of spontaneous or induced activity in normal populations is a highly important issue, which could affect interpretation of results from tinnitus studies. For evoked responses using EEG at least, these have been described comprehensively (192, 193) but the picture is less clear for induced or spontaneous oscillatory activity. In the visual domain, Muthukumaraswamy et al. (86) examined the degree of inter- and intra-individual variability and reproducibility of induced gamma-band oscillations following presentation of a simple grating stimulus. They discovered relatively large amount of inter-individual variability in frequency bandwidth and amplitude of gamma responses despite using a stimulus with highly optimized parameters. Their results indicate the unpredictability of gamma oscillations between people, suggesting that between-group studies on gamma oscillations is difficult, at least in the visual domain. Similar investigations of the amplitude and bandwidth of oscillatory activity are required in the auditory domain with non-clinical populations before any effects can be reliably attributed to tinnitus. Similarly, the stability of spontaneous oscillatory activity recorded weeks apart in non-tinnitus controls needs to be examined before any observed changes in brain activity of tinnitus patients can be reliably attributed to a specific course of treatment, such as specific acoustic sound therapies.

Given the variability between patients in clinical and non-clinical characteristics, it is likely that recorded oscillatory brain activity is also non-uniform. This individual variability in tinnitus patients should be reported as far as possible in an attempt to link specific individual characteristics to specific patterns of brain activity. Unfortunately, only a few studies have reported individual rather than averaged results for groups (149, 162).

One possible source of variability is the state under which data are collected, such as eyes open or closed, or while participants are watching a silent video. Cantero et al. (194) have shown that the properties of the neuronal circuits responsible for generation of alpha oscillations are modulated by the behavioral state. Eye closure introduces significant widespread alpha activity in the data, which can be misinterpreted for tinnitus-related activity, and can be avoided if data are collected with eyes open. Changes in oscillatory brain activity have also been shown to reflect states of normal arousal and cognition [e.g., Pfurtscheller and Lopes da Silva (36)] and numerous functional imaging studies using PET and fMRI have shown that sustained attention is associated with activation of a number of areas, mainly the prefrontal and parietal cortices (195–198).

The same cautionary note applies to studies of brain connectivity in tinnitus that rely on statistical correlations of oscillatory activity between disparate regions of the brain. Jin et al. (199) investigated the reliability of network metrics of functional connectivity networks of MEG covering the whole brain at the sensor level. They found that while the resting-state network was more reliable for eyes-open trials compared to eyes-closed trials in the alpha band (8–13 Hz), changes in functional connectivity network were the dominant feature in this frequency band. Furthermore, the gamma-band (30–45 Hz) networks were less reliable than the theta, alpha, and beta networks.

Attention can indeed have a strong impact on the frequency of ongoing activity. Studies with non-clinical participants have shown that alpha band activity in particular is gradually decreased as a function of attention load. In an EEG study, Boiten et al. (200) showed that the pre-stimulus level of alpha power is sensitive to the subject’s activation state. Their results indicate that when participants are required to perform under pressure or demand, alpha power is suppressed even when no stimulus is presented. Dujardin et al. (201) showed that event-related alpha (9–11 Hz) power decreased further for tasks that required high attention compared to tasks that required low attentional load. Potentially, the influence of auditory attention on oscillatory activity will be stronger in people with tinnitus compared to non-tinnitus controls. Behavioral studies have shown that chronic tinnitus has a detrimental effect on tasks that require attention, such as the Stroop task (202, 203). Therefore, the effect of attention on cortical dynamics must be disentangled from the tinnitus-related activity.

Another important factor is the existence, and the degree of hearing loss in tinnitus and it is not known how hearing loss alone influences cortical oscillations or the dynamic interaction between brain regions. In the MEG study by Ortmann et al. (158), the strongly right-lateralized gamma activity did not reflect tinnitus percept but rather the high frequency hearing loss that were particularly pronounced in the left ear. In our MEG study (149), we found that the increased delta activity was present in equal measure in the auditory cortex of most tinnitus participants, with and without clinically normal audiograms. We concluded that the abnormal slow-wave activity reflects tinnitus percept itself. These results suggest the possibly that different frequencies of oscillatory activity reflect different abnormalities in participants with tinnitus. We also concluded that the clinical definition of normal hearing may not be relevant to tinnitus for various reasons. First, the clinical audiogram does not account for possible existence of increased thresholds at intermediate frequencies (204); second, clinical audiometry allows for 20 dB hearing loss, which can contribute to the appearance of tinnitus; and third, increased thresholds at much higher frequencies (>8 kHz) are not measured, which can also underlie the appearance of tinnitus. The close association between tinnitus and hearing loss makes this a difficult topic to study but future research should examine the effect of the different degrees of hearing loss in the absence of tinnitus on oscillatory activity.

While diverse clinical characteristics may differentially affect brain oscillations and networks, certain non-clinical factors can affect these too. For example, electrophysiological activities in the brain may vary depending on the age group (27). Studies report a clear association between oscillatory activity and healthy aging, showing decrease in slow-wave activity in older healthy participants compared to younger ones (205, 206).

While differences between MEG and EEG can potentially underlie some of the discordant results, other issues related to data acquisition and differences between analysis techniques can be potential causes of the inconsistent findings. For example, if two independent studies use EEG to examine spontaneous activity in tinnitus participants, it does not necessarily mean that they should reach the same results, even if one assumes the virtually hypothetical situation whereby the participant groups from both studies are completely homogeneous. The understanding and analysis of convoluted M/EEG data requires extensive expertise and knowledge of countless issues (including neural sources, physical properties of signals, brain anatomy and physiology, source reconstruction, time series analysis, and statistics). The ways in which the data were acquired and treated, including different pre-processing steps and artifact rejection, down-sampling or filtering must be noted.

Different analysis approaches treat the data differently and may assess different aspects of the same data. This includes source localization techniques and their differing assumptions about the sources of recorded signals. These can potentially contribute to differences in the reported findings and so comparison between studies should be done with caution. Unfortunately, there are currently no standardized tools for analysis of M/EEG data and many labs make use of software they have created, which may not have been rigorously tested or validated in comparison to other existing techniques (207). For good practice and to minimizing certain operator related biases and discrepancies in data acquisition and analysis, researchers should attempt to abide by good practice guidelines as far as possible. For EEG, Picton et al. (208) cover topics related to experimental design, and analysis of event-related potentials, some of which are also relevant to MEG. For MEG, Gross et al. (207) provide extensive guidelines on data acquisition and analysis steps and suggest details that should be specified in manuscripts reporting MEG studies. These guidelines can strengthen the reliability and quality of research with M/EEG in tinnitus.

The heterogeneity of tinnitus is a major reason for inconsistent results, not only of M/EEG studies but also other neuroimaging and clinical trials. This is also a major obstacle in the development of effective therapies. There is an ever-increasing necessity for clearly defining subgroups of tinnitus patients based on multitude of factors including clinical and psychoacoustic characteristics and etiology. In an effort to better understand the heterogeneity of tinnitus, a pan-European Cooperation in Science and Technology (COST) program has recently been launched promotion a multidisciplinary tinnitus research network (TINNET) (http://tinnet.tinnitusresearch.net/). The aim of this action is to identify pathophysiological and clinically meaningful subtypes of tinnitus, which is essential in developing new treatment approaches and requires collaboration between scientists and clinicians. One specific focus of this network will be on developing standards for neuroimaging studies, including M/EEG and large-scale analysis of data from different laboratories in order to understand tinnitus-related changes in brain activity. Given the already complex M/EEG data, the identification of subgroups that are as homogeneous as possible will be a major step toward a more reliable evaluation of oscillatory changes in tinnitus.

## Conclusion

M/EEG techniques have progressed considerably from the early years of assessing evoked responses from the auditory cortex. Recent technological and methodical advances allow us to perform far more thorough research with focus on multiple brain regions concurrently to examine tinnitus-related spatial and temporal characteristics of oscillatory activity simultaneously from the entire brain. Quantitative connectivity measures based on coupling of spectral components of neuronal population activity may provide new insights into the mechanisms underlying brain organization in healthy and abnormal states. The possibility of assessing brain interactions on a network level is particularly exciting.

Despite numerous studies examining the neural mechanism of tinnitus, there is still considerable uncertainty regarding the changes in brain function and the structures involved in its processing. The main strength of M/EEG is that they allow direct observations of neural activity from human tinnitus sufferers in time and frequency. Their main limitation is their relatively poor spatial resolution, although these are constantly improving with the introduction of novel analysis techniques. While the detection, and mainly localization, of deeper sub-cortical sources remains a challenge for M/EEG, significant advances have been made to improve the source localization ability of these techniques. Differences between MEG and EEG data may partly explain some of the discordant finding in the tinnitus literature and simultaneous utilization of both techniques where possible will provide complementary and valuable information.

Coupled with subjective behavioral measurements from tinnitus patients, M/EEG provide unrivaled means of investigating the central mechanism of tinnitus. Based on some encouraging results to date, M/EEG are poised to contribute to our understanding of the neural abnormalities of tinnitus. With tinnitus the devil resides in the detail. To this end, high quality, well-controlled studies with clear hypotheses are required, which employ analysis techniques optimal for the specific type of data that are collected. One must be mindful to avoid the pitfall of misinterpreting M/EEG results, or reporting results that appear significant but are in fact physiologically and technically improbable, or at least are unrelated to tinnitus. New and existing results require constant verification from different research groups. No single finding should be taken for granted but each should be evaluated with respect to other studies that do not report the same phenomena and differences between results from different groups should be at least discussed. Finally, when interpreting novel findings, it is better to be cautious than impulsive, and therefore findings from M/EEG studies have to be evaluated with due consideration to the limitations of these techniques and the analysis methodology employed.

## Conflict of Interest Statement

The author declares that the research was conducted in the absence of any commercial or financial relationships that could be construed as a potential conflict of interest.
